# Music in the Morningside (Edinburgh) Lunatic Asylum

**Published:** 1843-01

**Authors:** 


### Music in the Morningside (Edinburgh) Lunatic Asylum.

On Tuesday evening Mr. Guynemer, one of Mr. Mainzer's professors, accompanied by several members of the Committee, and met by some of the Managers of the Asylum, held a *séance* in the new building. Dr. M'Kinnon, the excellent superintendent, who had made all the requisite preparations, and provided the music-books, assured the gentlemen, when they arrived, that his patients, above eighty well-dressed patients, nearly equally of each sex, were all delighted with the novelty, and were actually met in the chapel, anxiously expecting the welcome strangers. One of the gentlemen addressed "*the meeting*," and in a friendly and familiar way introduced the subject and the professor, giving a brief history of Mr. Mainzer and his wonderful exertions and success, and stating the wish of the Committee that Morningside should have a share of what was making everybody in Edinburgh so happy. He was listened to with fixed attention, and applauded, according to established custom. At Mr. Guynemer's request, the audience gave a specimen of their Sunday psalmody by singing the hundredth psalm. This they did as well as most, and greatly better than many congregations. Every one present heartily joined. Encouraged by this trial, he proceeded to bring out the fundamental note *sol*, which, after an effort or two, was sung correctly by nearly the whole audience, and in a sensible business-like way, with none of that tittering which marks *sane* pupils. *La* and *si* followed, sung sometimes *forte*, sometimes *piano*, now by the ladies alone, then by the gentlemen, and now by both. Chords were achieved, the ladies singing one line and the gentlemen another in harmony, with this difference, when compared with *extra-mural* classes, that it was more readily, steadily, and *unerringly* done by the *intras*. The pupils were examined on the lines, spaces, and names of notes, and numerous voices called them out correctly; all were intent on their books, and had no difficulty in reading the right numbers of the short exercises, as well as, guided by the eye, singing the right notes. When three notes had been mastered, Mr. Guynemer, who conducted the lesson with uncommon tact and spirit, showed what three notes could be made to do, by singing a song of uncommon beauty (composed by Rousseau), and with his instrumental accompaniment, fine voice, and science, produced a manifest effect on the audience: who showed their capacity to distinguish really good music by expressing the pleasure they felt by murmurs of approbation while he sang, and a hearty round of applause when he concluded. The first lesson concluded with No. 15 of the well-known book, and a hymn of Mr. Mainzer's sung in parts, at sight, by several of the attendants, who volunteered, led and accompanied by Mr. Guynemer. The patients left the hall with the same decorum which they had preserved throughout; there not having been, to our purposely-watchful eyes, a word, a look, or a movement, which could have led a stranger, unconscious of the place, to discover where he was

Practical conclusions rush into the mind from the experiment just described, too numerous and too weighty to be here indulged in. Every one may be expected to exclaim—what a commentary upon the enlightened view now taken of that merely physical ailment, that no longer mysteriously awful visitation, called insanity!—what a commentary upon that kindly, encouraging, "ministering to the mind diseased," which, so long ridiculed and dreaded, it is now a deep disgrace to any asylum not to follow! We are struck with this proof of the possibility of concentrating a large assemblage of mental patients in one safely-exciting pursuit. During the sitting not one of them dreamed of his or her peculiar extravagancies; *that* portion of the brain of each was quiescent, and the more and the longer quiescent, like fair play to a sore, the more likely and the more speedy will be the cure.

The friendly visit of Mr. Guynemer, and the "gentlemen," its respectful and evidently welcome reception, is also an instructive feature, strongly contrasted with the old abandonment of "the maniac," as he was called with a shudder, to chains and straw. There was not a patient present, probably, who did not feel that this was a recognition of his still belonging to society, and being the object of the sympathy and brotherly love of his fellow-men who are blessed with health of mind, and are actively and benevolently busied with the best means of restoring the same blessing to himself. Visits of strangers from mere curiosity are to be deprecated; but visits such as this to a well-conducted asylum may have the very best effects on the inmates. Fears to mingle with the insane there might be in the irritation of the old coercive system; there can be no such fears under the new. No stranger present, we are assured, would have hesitated to have sat down even unattended among these eighty pupils of Mr. Guynemer.—*Caledonian Mercury.*

